# A Lordly Fraud in Texas

**Published:** 1886-02

**Authors:** 


### A LORDLY FRAUD IN TEXAS.

The "Empire of Texas" has not been wont to welcome invaders in a hospitable manner. She has been proud of her Alamos and her San Jacintos; and, doubtless, many of those who, in former days, essayed the conquest of Texas, carry to this day mementoes of their reception.

But times have changed, and we have changed with them, as the proverb goes, it seems. Texas has been captured! has surrendered! Not to an "army with banners" (which, according to the Bible, was regarded as the most dreadful thing); not with the pomp and circumstance of "war's magnificently stern array"; but invaded, raided and captured by as magnificent an exponent of the *genius of humbug* as ever dazzled the eyes, or emptied the purses of an exceedingly gullible community. He travels in a Pullman Palace car, with all the accessories and emblazonry of Royalty. Not Pompey's triumphant pageant, with his captives at his chariot wheels, was grander, more pompous and imposing; nor were his captives more captive than the simple souls who surrendered to this conqueror.

His name was Flower; his hame—ah, go ask of "ye winged winds!" "Doctor" Flower! a magnificent blossom on the stem of a luxuriant and perennial IMPUDENCE—from away up in the land of the righteous; a bloom from the hot-houses of humbuggery! He came down on us like the wolf on the fold; or, to use a more expressive, if less elegant figure, like a thousand of bricks! Like Cæsar, he *veni-d*, *vidi-d*, *vici-d*—and got! It was no little faded flower, however fondly dear; not a bit of it. It was a veritable sunflower of its kind; yet some there be who will hold him ever "in memory dear." As the perfume is to the rose, even so was *genius* to this Flower. He was chuck full of it; it was exuberant and abundant. Moreover, it asserted itself successfully."

Lord Bolinbroke assures us that "it (genius) is the same principle that gives inspiration to the poet, conception of beauty to the artist, brilliancy of argument to the advocate, as well as ingenuity to the midnight burglar, and the common swindler." This blossom was at once poet, artist and advocate, in his line of business, but, so far as we know, he was *not* a "midnight burglar." He was not even a night-blooming cereus. Nor was he a "common swindler;" far from it. He was the most uncommon swindler that ever astonished the natives in this g-a-lorious country! No sir! He disdained the vulgar method of the common herd of tricksters. He was to them what the eagle is to the sparrow hawk. No tom-tit of a ten-dollar fee for him! He swept majestically down from his Pulman-Palace eyrie, and incontinently gobbled up his five hundreds and his thousands, with an ease and grace that would have done credit to Tom Ochiltree in his palmiest days at poker. It *takes genius* to do that. Oh! he was a magnificent fraud.

It is said on good authority that a well-known citizen of———

who had been told by a learned and experienced physician that he had organic disease of the heart, and could not be cured, went to this fellow. The "Doctor" assured him of a speedy cure, and demanded $500, which, strange as it may seem, was paid on the spot. All the victim got was a prescription. When asked if he would guarantee a cure, this lordly cuss smiled sweetly, and said, "Yes, come to Bosting and I will guarantee to cure you, but my fee will be ten thousand dollars." Thus the victim did not get "a guarantee," but he got an "assurance" (the Doctor has some left), and a prescription, and is happy,—but not well,—it is needless to remark.

Hide now your diminished heads, oh, ye disciples of Galen and of Hip! your occupation's gone. How can you consent to jog along the winding path of mediocrity, now that *genius* has gone ahead, and emblazoned and illumined a royal road to fortune and to fame! *Vive la bagatelle*!

